# Large language models for promoting physical activity: a review of experiential and behavioral outcomes, social roles, and human-likeness in persuasive LLMs

**DOI:** 10.3389/fdgth.2026.1869793

**Published:** 2026-07-08

**Authors:** Alessandro Silacci, Arianna Boldi, Maurizio Caon, Amon Rapp

**Affiliations:** 1 Digital Business Center, School of Management of Fribourg, HES-SO University of Applied Sciences and Arts Western Switzerland, Fribourg, Switzerland; 2 Computer Science Department, University of Turin, Torino, Italy

**Keywords:** anthropomorphization, behavior change, conversational agents, large language models, physical activity

## Abstract

Large Language Models (LLMs) are rapidly reshaping the landscape of conversational agents for health behavior change, enabling more human-like interactions than earlier rule-based systems. In the domain of physical activity promotion, however, the understanding of the outcomes of these technologies remains fragmented. This review examines the current state of LLM-based conversational agents designed to support physical activity, drawing on 13 studies. The analysis identifies three cross-cutting themes. First, it emphasizes the experiential, motivational, and behavioral outcomes of the reviewed studies, highlighting positive effects on user engagement, while stressing that evidence for a direct, sustained impact on objectively measured physical activity remains limited. Second, it shows that LLMs may assume a variety of social roles, entailing different relational dynamics. Third, it points out that people anthropomorphize LLM-based conversational agents, which can enhance emotional investment and strengthen the user-agent “relationship”, but may also foster over-reliance and misplaced expectations. Building on these findings, we critically discuss ethical concerns raised by the growing persuasive capacities of LLMs in this domain, including the redistribution of agency between users, technologies, and third parties and the risks tied to users’ tendency to ascribe humanness to artificial agents.

## 1 Introduction

Conversational agents, namely, software systems that interact with users through natural language, are increasingly being incorporated into digital health interventions to encourage health-related behavior change (1). In areas such as physical activity (PA) promotion, these agents have demonstrated encouraging outcomes by increasing user involvement, facilitating self-management, and promoting sustained adherence. Typically, they are designed to resemble human conversation, delivering educational content (2), stimulating self-reflection (3), and helping users progress toward their behavioral objectives (4).

Nevertheless, earlier generations of conversational agents, like rule-based agents, which follow predefined scripts and decision rules, often struggled to produce fluid and responsive interactions, frequently resulting in user dissatisfaction due to their inflexibility and overly rigid structure (5). The advent of Large Language Models (LLMs), AI systems trained on large text corpora to generate human-like language, represents a major evolution in the effectiveness of conversational agents for behavioral purposes (6). These models support more flexible and natural exchanges, produce human-like dialogue flows, and generate context-aware replies (7–9), all of which can significantly strengthen the impact and accessibility of behavior change interventions. Preliminary evidence shows that LLM-based conversational agents are capable of reproducing elements of counseling practices (10). Such capabilities relate to long-standing priorities in Human-Computer Interaction (HCI) research, particularly the development of systems that can support people in enhancing their own well-being through relevant and personalized human-like advice (11–13).

A picture of the early literature in this domain has been provided by Silacci et al. (14), highlighting that LLM-based conversational agents have demonstrated promise for supporting physical activity, yet challenges persist, including reliance on proprietary models with limited reproducibility. However, Silacci et al.'s review is limited to early works, and a comprehensive understanding of user experience of LLMs in supporting physical activity is still needed.

To address this gap, this mini-review aims to outline studies addressing the user experience of LLM-based conversational agents for physical activity promotion, guided by two questions: (i) how do users experience these agents? and (ii) what relevant consequences follow from their capacity to assume specific “social roles” and display human-like qualities? As a mini-review, it provides a focused, narrative synthesis rather than the exhaustive coverage of a systematic or scoping review. It thereby makes two contributions: it maps the current landscape of this rapidly developing area, and it critically discusses how the design of “persuasive” LLM-based conversational agents, namely, agents which have the main goal of modifying people's behavior, may raise new ethical concerns.

The article is structured as follows. Section 2 outlines the method used to select and review the papers, whereas Section 3 presents the findings of the review. Section 4 concludes the article by discussing these findings, introducing relevant ethical themes, highlighting the review's limitations, and outlining directions for future research.

## 2 Method

Given the rapid evolution of LLM-based conversational agents and the limited number of available studies, a mini-review was considered the most appropriate format to provide a timely synthesis of emerging evidence. To identify relevant studies, keywords related to conversational agents and physical activity were derived from preliminary literature reviews on conversational agents for wellbeing (14, 15). Searches were performed in the Clarivate Web of Science (WoS) and Elsevier Scopus, two databases recognized for their comprehensive coverage of peer-reviewed academic research (16, 17), including ACM and IEEE proceedings, which are central to HCI and AI research, and were deemed sufficient for the streamlined scope of a mini-review. The search strategy was developed, refined, and executed solely by the authors (A.S., with input from all co-authors); no academic librarian or information specialist was involved in its design or validation. Queries included papers from November 2022 (as this marks the introduction of ChatGPT, and the diffusion of LLMs) to April 2026. They combined keywords pertaining to Agents and to Physical Activity.

Records were deduplicated using a custom Python script and managed in a spreadsheet; no dedicated screening platform was used. One reviewer (A.S.) screened all records against the inclusion criteria, first by title and abstract and then by full text and also performed data extraction and quality appraisal. These processes were not independently checked by a second reviewer. Papers were included if:

i) they were published in peer-reviewed international conference proceedings or journals between November 2022 (which corresponds to the public launch of ChatGPT and the broader diffusion of LLMs to the general public) and April 2026;
ii) if they presented primary research on the design, development, and/or evaluation of an LLM-based conversational agent for the purpose of supporting physical activity;
iii) and if they reported an empirical study with human participants.

Conversely, works were excluded if:

i) they were written in a language other than English;
ii) if they were grey literature, preprints, unpublished or under-review manuscripts, commentaries, opinion papers, position papers, study protocols, conference abstracts, abstract-only submissions, books, or book chapters;
iii) or if they did not present an empirical study with human participants.

**Figure 1:**
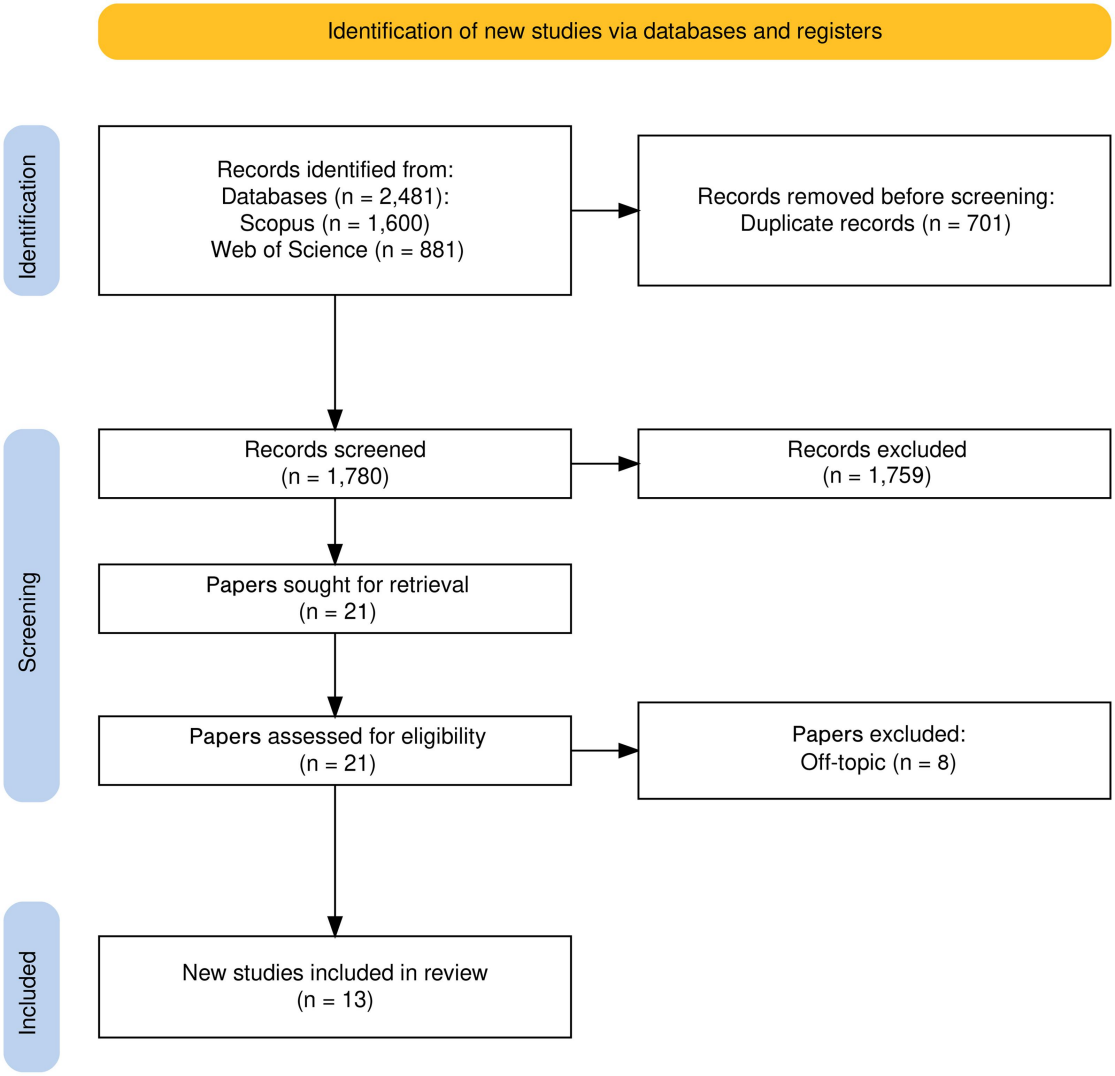
The PRISMA flow diagram illustrating the steps taken for the paper selection.

Screening, data extraction, and quality appraisal were performed by the first author. The search identified 2,481 records across Scopus and Web of Science. After removing 701 duplicates, the remaining records were screened by title and abstract; 21 articles were then assessed at full text, and 13 peer-reviewed articles met the inclusion criteria (Figure 1). For each included study, we extracted the LLM's usage and role, the study goal, sample, experimental design, and main findings (Table 1). The extracted data were synthesized narratively using thematic analysis (18): the three cross-cutting themes presented below were developed iteratively, combining inductive analysis of the included studies with the guiding focus on review's goals, and were refined through discussion among the first and the last authors. Quality appraisal of the included studies was performed using Mixed Methods Appraisal Tool (MMAT) criteria (19).

**Table 1:** The papers included in this review.

Author, year	Location	LLM Role	Sample	Sample Type	Study Design	Theme(s)	Key findings and evidence type	Quality appraisal (MMAT)
Sun et al., 2024 (20)	Netherlands (implied, not stated)	Coach	*N* = 66	End-users (adults age 18–65)	Quantitative	- Primary: Experiential & behavioral outcomes (PA change, engagement, motivation). - Secondary: Social roles (coach role).	- Humorous chatbot increased step counts vs. both non-humorous and control → statistically significant, objective - Non-humorous vs. control: no PA difference → null - Engagement mediated the style→PA effect (EMA) → statistically significant, self-report - Motivation: no effect, no mediation → null	An adequately powered small three-arm RCT with an objective step-count outcome and a clean engagement-mediation result, but with reporting gaps on randomization, baseline balance, and attrition.
Strömel et al., 2024 (23)	Not stated	Assistant	*N* = 283	End-users (adults 10: M = 27.7, SD = 3.8; 273: M = 43.16, SD = 12.75)	Mixed	- Primary: Experiential & behavioral outcomes (reflection, engagement). - Secondary: Social roles (assistant role).	- Formative interviews on tone, abstraction, and distrust of the “black box” → qualitative - LLM text vs. charts: no effect on overall reflection (null), but higher comparison, focused attention, and reward → statistically significant, self-report - Open-ended sensemaking responses → qualitative	A well-integrated sequential design with a large quantitative sample, though the primary reflection effect was largely null (one subscale significant), so reflection claims should be stated cautiously.
Wachholz et al., 2025 (33)	Austria	Recommender System	*N* = 119	End-users (adults: M = 28.5, SD = 7.4), Coaches (adults)	Mixed	- Primary: Social roles (recommender) - Secondary: Experiential & behavioral outcomes (trust, acceptance/TAM), Human-likeness (coaches unable to distinguish AI- vs. human-made plans).	- AI-tool users reported higher trust, usefulness, and intention than non-users (user-friendliness null; only 16 AI users) → statistically significant, self-report (observational) - AI plan rated more trusted than the human plan overall (null among non-users) → statistically significant, self-report - Coaches preferred the AI plan and judged the human plan too detailed; 4/6 identified the AI plan → expert evaluation (descriptive)	An exploratory pilot whose small, non-representative samples (only 16 AI users; 6 coaches) and limited qual-quant integration make findings suggestive rather than confirmatory.
Imai & Misaki, 2025 (21)	Japan (implied, not stated)	Coach	*N* = 14	Lay raters (young adults 21–25), End-users (young adults 21–24)	Mixed	- Primary: Experiential & behavioral outcomes (engagement, cognitive load, body-data connection). - Secondary: Social roles (coach role).	- Voice narrative beat text on aesthetic appeal (reward null) → statistically significant, self-report - Gesture interaction reduced frustration vs. tap (performance null) → statistically significant, self-report - Most participants reported stronger bodily connection with voice/gesture → subjective perception (descriptive)	Very small early-stage crossover pilots (*N* = 10 each) with isolated single-comparison effects and an under-specified qualitative component, best read as preliminary signal.
Jörke et al., 2025 (25)	USA	Coach	*N* = 16	End-users (adults age 21–71)	Mixed	- Primary: Social roles (coach role, MI-based facilitative coaching). - Secondary: Experiential & behavioral outcomes (perceived support, personalization) and Human-likeness (conversational rapport).	- Formative and lab-study interviews → qualitative - Survey: felt supported, comfortable sharing, advice personalized (descriptive means) → subjective perception (not inferentially tested) - Independent MITI coding: ∼93% MI-consistent/neutral, better than vanilla GPT-4 → expert evaluation - Inconsistent use of wearable data → expert/qualitative-noted limitation	A methodologically careful qualitative and expert-coded (MITI) study whose quantitative survey is descriptive and small-N, so it evidences feasibility and experience rather than effectiveness.
Shin et al., 2025 (27)	South Korea	Coach	*N* = 18	End-users (adults age 19–54)	Mixed	- Primary: Experiential & behavioral outcomes (usefulness, usability, plan quality). - Secondary: Social roles (planner/coach role).	- Formative interviews → qualitative - Survey: high perceived usefulness and ease of use (descriptive) → subjective perception - Generated plans met ACSM/US guidelines (intrinsic check) → rule-based/expert check - Expert raters judged plans sound on FITT, weaker on exercise-type variety → expert evaluation	A well-triangulated (user + intrinsic + expert) evaluation that is nonetheless single-session, small-N, and descriptive, supporting feasibility and plan quality but not behavioral efficacy.
van Arum et al., 2025 (28)	Not stated	Recommender system	*N* = 124	End-users (adults age 18–62)	Mixed	- Primary: Social roles (AI as collaborative partner vs. trusted human advice sources). - Secondary: Experiential & behavioral outcomes (trust calibration) and Human-likeness (anthropomorphism of MoveAI).	- Trust in MoveAI fell below medical professionals but matched trainers/dietitians/GPs and exceeded friends/family → statistically significant, self-report (with honest nulls vs. several human experts) - Trust correlated with intuitive/dependent decision styles (avoidant null) → statistically significant, self-report (correlational) - Interview themes: selective/conditional trust, “generic” advice, anthropomorphism → qualitative	A well-designed convergent mixed study with a good sample, but it captures trust attitudes toward a simulated scenario interaction rather than deployment or behavior.
Haag et al., 2025 (22)	United Kingdom	Recommender system	*N* = 58	Experts (adults), Lay raters (adults)	Quantitative	- Primary: Social roles (AI vs. layperson vs. healthcare-professional as intervention author). - Secondary: Experiential & behavioral outcomes (rated appropriateness/effectiveness/engagement).	- GPT-generated messages rated more appropriate, engaging, effective, and professional than both layperson- and HCP-authored → statistically significant: expert evaluation (HCP raters) and subjective ratings (laypersons) - Layperson- vs. HCP-authored: mostly no difference → null - No objective PA outcome, no real patients: findings reflect rated message quality only	An internally rigorous rating study with appropriate mixed-model analysis, but limited external validity: no patients and no behavioral outcome, only rated message quality in hypothetical scenarios.
Li et al., 2025 (32)	Taiwan (implied, not stated)	Coach	*N* = 18	End-users (unspecified)	Quantitative	- Primary: Social roles (coach) - Secondary: Experiential & behavioral outcomes (reflection, insight).	- System beat baseline on reflection, exploration, and insight → statistically significant, self-report - No effect on self-reflection ability, and no significant LLM increment over visualization alone → null - Participant comments on understanding and prompt style → qualitative/subjective perception	A small early-stage within-subjects study showing significant reflection effects that are self-report only and single-session, with clear nulls on reflection ability and on the LLM increment.
He et al., 2026 (26)	Not stated	Recommender system	*N* = 5	Experts (adults age 18–45)	Quantitative	- Primary: Experiential & behavioral outcomes (plan quality/personalization).	- Outperformed baselines on ranking and plan-quality metrics, including a simulated “user satisfaction” score → computational benchmark (no human inferential test) - Ablations (knowledge graph, RLHF, dynamic profiling) → descriptive computational - 5 expert trainers rated the system safer than a general LLM → expert evaluation (descriptive)	An offline engineering benchmark rather than human-subjects evidence, providing no end-user behavioral or experiential data and reporting “user satisfaction” only as a simulated metric without inferential tests.
Hoang et al., 2026 (24)	Not stated	Coach	*N* = 140	End-users (adults age 18–55+)	Mixed	- Primary: Social roles (MI coach; motivation- vs. planning-focused strategy). - Secondary: Experiential & behavioral outcomes (readiness, intrinsic motivation).	- Readiness to change rose after interaction in both conditions → statistically significant, self-report (within-group, not an LLM advantage) - Primary between-condition predictions (readiness gain, perceived MI, working alliance) → null - Reaching a behavioral-plan agreement was associated with greater readiness gain → statistically significant, self-report (exploratory) - Agent MI adherence assessed by automated GPT-4o coding → computational (not human expert evaluation) - User feedback themes → qualitative	A reasonably sized hypothesis-testing study whose primary between-condition predictions were null, leaving the positive findings resting on a within-group readiness rise and exploratory behavior correlations.
Jörke et al., 2026 (29)	USA (implied, not stated)	Coach	*N* = 158	Experts (adults), Lay raters (adults), End-users (adults)	Mixed	- Primary: Experiential & behavioral outcomes (PA, beliefs, enjoyment, engagement). - Secondary: Social roles (LLM coach Beebo) and Human-likeness (relational cues).	- Both conditions increased objective PA vs. baseline; no LLM advantage over the no-LLM control → statistically significant, objective (within-group) + null between conditions - LLM group reported stronger positive mindsets (enjoyment, self-compassion, belief in benefits) → subjective perception/qualitative (not inferentially tested) - LLM group showed much higher app engagement and retention → objective usage logs (descriptive)	An honest, well-integrated formative RCT with objective and qualitative data that is deliberately underpowered for between-condition PA, making its contribution exploratory and mechanistic.
Silacci et al., 2026 (30)	Switzerland	Companion	*N* = 280	End-users (young adults M = 21.2, SD = 2.66)	Mixed	- Primary: Social roles (AI peer vs. human peer vs. control; working alliance, social presence). - Secondary: Experiential & behavioral outcomes (steps, relatedness, competence) and Human-likeness (human vs. cyborg avatar, believability).	- Human peers logged more steps than the AI-cyborg peer; no overall PA increase vs. control → statistically significant, objective (with major nulls) - AI peers formed stronger working alliances than human peers → statistically significant, self-report - Human peers evoked higher social presence than AI peers (against prediction) → statistically significant, self-report - Relatedness and competence → null - Interview themes (human accountability vs. steady AI support) → qualitative	Rigorous design with long-term, four-arm, randomized, with an objective behavioral outcome and validated scales, though high attrition (54%) and a largely null behavioral result vs. control temper the behavioral (not the relational) conclusions.

To support transparency and reproducibility, the full search strings are openly available in an OSF repository.^1^

## 3 Results

Drawing on 13 studies, the review outlines how LLM-based conversational agents can be used to promote physical activity.

Across the 13 studies, research designs were predominantly mixed-methods (*n* = 9, ∼69%) or quantitative (*n* = 4, ∼31%), with no purely qualitative study identified. Sample sizes were generally small (median *N* = 66, range 5–283), with five studies (∼38%) enrolling 22 or fewer participants and six studies with samples of N ≥ 100 (∼46%) being confined to online surveys or field deployments. Studies were conducted in Europe (*n* = 4, ∼31%), Asia (*n* = 3, ∼23%), and the USA (*n* = 2, ∼15%), with the remainder not reporting their location; participants were mostly healthy, digitally engaged adults, complemented in some studies by experts or lay raters. Outcomes were chiefly self-reported or based on expert ratings, with only three studies collecting objective physical activity data (∼23%), and only two reporting longitudinal deployments with follow-up (∼15%).

The review is organized around three themes: i) the experiential, motivational, and behavioral outcomes of LLM-based conversational agents, ii) the social roles they assume, and iii) the human-likeness of the interaction. Table 1 offers a comprehensive overview of the studies included in this review.

### 3.1 Outcomes of the interventions

To provide a comprehensive overview of the outcomes of the reviewed studies, we focused on i) user experience outcomes, such as user engagement, ii) motivational outcomes, like readiness to change, iii) content quality (i.e., the perceived quality of the output provided by the LLM), iv) and behavioral outcomes of the interventions.

User engagement was frequently reported as a key outcome of the proposed design solutions. Sun et al. (20) identified design elements such as humor as potential enhancers of user engagement. Imai and Misaki (21) also suggested that multimodal delivery of the intervention may deepen engagement. Similarly, Haag et al. (22) demonstrated that LLM-generated just-in-time adaptive interventions are rated significantly higher than human-crafted alternatives in terms of engagement. Strömel et al. (23), instead, found that users may experience more refection, focused attention, and reward when presented with LLM-generated qualitative physical activity data compared to standard charts alone. These findings, however, derive from markedly different designs, sample sizes, comparators, and outcome measures, ranging from a controlled trial with objective behavioral data to a rating study of message quality without end-user deployment.

As for the motivational outcomes, Hoang et al. (24) assessed readiness to change before and after interaction with a motivation-aware LLM coach vs. a baseline agent: both conditions produced significant within-group increases. Jörke et al. (25) implemented an LLM-based physical activity coaching chatbot grounded in motivational interviewing (MI), reporting positive user adherence to MI strategies.

With reference to the quality of LLM output, some research has focused on aligning LLM outputs with established guidelines and expert knowledge. He et al. (26), for example, developed and validated a purpose-built system for generating personalized, science-driven training plans. Shin et al. (27) proposed a pipeline integrating expert-verified exercise lists and global guidelines to improve alignment with real-world practice. Van Arum et al. (28) showed that while initial LLM recommendations may tend to be generic, they can become progressively more personalized through iterative user input.

As for behavioral outcomes, Jörke et al. (29) evaluated an LLM-augmented coaching app against a no-LLM control, and observed that both conditions significantly increased objectively measured physical activity relative to baseline; however, no clear advantage for the LLM condition emerged in the short-term, with the authors suggesting that LLM coaching may more readily shift mindsets and motivational precursors than immediate behavior. In a six-month trial, Silacci et al. (30) compared human peers and LLM-driven simulated exercising peers, finding that human dyads maintained significantly higher step counts than AI peers across deployment and follow-up.

That said, only a minority of the reviewed studies explicitly linked agent interactions and features with objectively measured behavioral outcomes, and only two (29, 30) did so over a sustained, longitudinal deployment; the remainder were single-session or cross-sectional. Taken together, these studies suggest that LLM-based interventions might influence motivational precursors to behavior, but evidence for direct, sustained effects on objectively measured physical activity remains limited and inconsistent.

### 3.2 LLM roles

LLM-based conversational agents may assume a range of social roles, which can be intentionally designed through personas and prompting strategies or emerge through interaction: these roles are characterized by specific behavioral patterns, relational expectations, and degrees of authority that shape how users perceive and engage with the system (31). Coaching roles (*n* = 7, ∼54%) dominate the reviewed literature (20, 21, 24, 25, 27, 29, 32), typically centering on directive guidance and motivational support. Additional roles include assistants (*n* = 1, ∼8%) (23), companions (*n* = 1, ∼8%) (30), and recommender systems (*n* = 4, ∼31%) (22, 26, 28, 33).

Coaches are primarily intended as proactive agents that provide personalized support by considering the qualitative aspects of a person's context, such as goals, values, preferences, and past experiences (25). Here, the LLM is a knowledgeable authority expected to deliver accurate, evidence-based guidance (34). This role may shift toward a deeper interpersonal dynamic, drawing on Motivational Interviewing as theorized by Miller and Rollnick (35, 36), a client-centered approach that guides individuals to explore and resolve ambivalence toward behavior change. For example, GPTCoach (25) follows a facilitative, non-prescriptive approach, adopting a supportive, non-judgmental tone. Similarly, Hoang et al. (24) designed a digital coach that adapts strategies to users’ motivational states. In the same vein, Bloom leverages qualitative context from coaching conversations to personalize a number of LLM-augmented behavior change interactions (29).

The remaining social roles assigned to LLMs can be better understood through distinct theoretical lenses. The assistant role positions the agent as a task-oriented aide, aligned with Luger and Sellen's (37) characterization of conversational agents as responsive tools whose effectiveness depends on bridging user expectations and system capabilities. The companion role, instead, emphasizes emotional presence over informational utility, and can be grounded in the Computers Are Social Actors paradigm (38, 39) which shows that users instinctively apply social scripts from human relationships to technology interactions. Bickmore and Picard (40) demonstrated that agents with deliberate relationship-building behaviors are liked more, trusted more, and retain users significantly longer than purely task-oriented equivalents. Finally, the recommender system role positions the LLM as a personalization engine, building on a long tradition of collaborative filtering and content-based approaches (41), now extended through conversational interfaces that enable more context-aware recommendation strategies.

The studies highlight different relational dynamics depending on the agent's role. When cast as a coach, as in GPTCoach (25) and Bloom (29), or Hoang et al.'s motivation-aware agent (24), the relationship takes on a vertical, asymmetric quality, in which the agent is positioned as an authoritative guide. When the role shifted toward a peer, as in Silacci et al. (30), the intended relationship is perceived as horizontal and co-experiential.

Across these framings, a consistent pattern emerges: users form relationships with LLM agents, valuing consistency, availability, and non-judgmental framing (29, 30, 32), while remaining skeptical of claims to authentic reciprocity (29, 30). What is strikingly absent, however, is any exploration of relationships beyond the dyad, whether through group facilitation, multi-user turn-taking, or collective tasks involving family or community members.

### 3.3 Human-likeness of the interaction

Recent research shows that users often perceive Generative AI systems not only as “tools” but also as “beings” endowed with a certain degree of agency, namely, the system's capability to operate with a degree of self-direction and proactivity, even though these systems have no internal understanding and merely simulate such qualities (42, 43). It comes as no surprise, therefore, that the human-likeness of LLMs’ conversational outputs and the processes of anthropomorphization enacted by users were central concerns in the reviewed literature. At its core, anthropomorphization refers to the process of ascribing humanness to entities, that is, the attribution of characteristics that define what it means to be human (44).

Jörke et al. (25), for example, found that many participants interacting with GPTCoach reported the experience as conversing with a human coach. However, an interview study with experts reported skepticism about the possibility of forming a deep relational connection with an AI. Similarly, van Arum et al. (28) pointed out that several participants involved in the evaluation study of MoveAI, a GPT-4.0-based conversational agent, anthropomorphized it using pronouns (i.e., he or she) when referring to it. Jörke et al. (29) found that many users involved in the evaluation of Beebo, an LLM-based conversational agent designed to act as a coach, explicitly said that chatting with Beebo felt like talking to a person, even while recognizing it was an AI. Participants identified several qualities that fostered this sense of presence, mentioning Beebo's empathetic and non-judgmental tone and its references to prior conversations.

These studies highlight the ease with which users may ascribe human-like qualities to LLMs and how these perceived qualities may affect the ongoing interaction. Jörke et al. (29) particularly stress both the benefits and risks of the LLMs’ relational cues, which may trigger the users’ ascriptions of humanness. On the one hand, they may help users maintain accountability and foster positive mindsets around exercise. On the other hand, such designs may foster emotional attachment or over-reliance on the chatbot.

The human-likeness of interaction also points to new human-like collaboration dynamics between humans and AIs (45). LLMs produce more natural, adaptive, and persuasive dialogue capabilities than previous rule-based technologies (1), enabling the creation of collaborative human-AI arrangements that may encourage personal growth (46). On this point, Silacci et al. (30) found that AI peers proved more effective at sustaining a reliable working alliance, as human connections were often experienced as authentic but inconsistent. LLM peers did not convey authenticity in the same way as humans, but they consistently provided encouragement, availability, and a non-judgmental stance. Hoang et al. (24) reported that users who reached agreement with agents on a change plan were more likely to experience greater increases in their intrinsic motivation to change.

These findings highlight that the relational and emotional consequences of perceiving human-like qualities in LLMs may have double-edged effects in the physical activity domain.

## 4 Discussion

### 4.1 Limited behavioral effectiveness, generalizability, and reproducibility

The reviewed studies suggest that LLM-based conversational agents may improve engagement, perceived personalization, reflection, and some motivational precursors, like readiness to change. However, evidence of a direct, objective, and sustained effect on physical activity remains limited and inconsistent.

Moreover, the studies included in this review largely relied on selected samples, often involving small participant groups, and were heterogeneous in terms of study designs and outcomes. The limited number and heterogeneity of these studies restrict the comparability of findings across studies and reduce the strength and generalizability of the conclusions that can be drawn from this mini-review.

Most evaluations were also conducted in controlled or experimental settings. Furthermore, this body of research has largely overlooked underrepresented populations, including older adults, individuals with low digital or health literacy, people with complex chronic conditions, socioeconomically disadvantaged groups, and people with physical limitations. As a result, the generalizability of current findings remains uncertain in this regard as well. The benefits observed among relatively homogeneous and digitally engaged participants may not translate to populations facing cognitive, physical, socioeconomic, or technological barriers to adoption and sustained use.

Another concern relates to the reliance on proprietary LLMs, which raises challenges for reproducibility. These systems, such as OpenAI's GPT series, are fundamentally nontransparent black boxes limiting the ability to replicate results (47). Moreover, interventions developed using specific model versions may be difficult to replicate as model behavior can evolve over time through updates that are not always documented (47). To mitigate these concerns, researchers should provide comprehensive documentation of the LLM configuration and deployment process, including the timing of model access, application programming interface (API) version information, and prompting procedures.

### 4.2 Transformation of relational roles

The social roles that LLM-based conversational agents can now assume point to several interactional concerns that need to be discussed.

The coach role implies an asymmetry of expertise, where the user may perceive LLMs’ authority as intrinsically reliable, which may lead them to mindlessly follow their advice and thus potentially provoke user harm. By contrast, the companion role may foster attachment, encouraging the user to invest in a “relationship” with an entity that cannot reciprocate (48). Similarly, the peer role may simulate relational horizontality and encourage the conception that LLMs resemble humans (49), which can distort moral judgments by fostering beliefs that LLMs can be held responsible for their actions, despite the fact that LLMs are not moral agents (50). Finally, the recommender system role raises issues of opacity and algorithmic personalization: LLMs operate without revealing the internal processes underlying their outputs (51). This opacity raises significant concerns regarding the extent of control that users can exercise over the interaction, posing important ethical questions regarding responsibility and autonomy: for example, who is responsible for the effects of a recommendation if it produces side effects or negative outcomes?

However, if we look beyond the empirical findings analyzed in this review, to the theoretical literature that has examined the role of AI in behavior change, we may see that instances of domination do not come only from the technology, but also from third parties that exert their power through it (52), like private subjects or public institutions which could use persuasive LLMs for their own ends, a risk that Lupton (53) has noted as already present in our society. Purpura et al. (54) note how, in the context of dieting and exercising, behavior change technologies might aim to enforce sublimated social goals, reinforcing conceptions of what it means to be healthy or fit. A relevant theoretical concern highlighted by previous literature, therefore, is that, as LLMs become more persuasive by acting as human experts or “friends”, they may gain greater authority and trust and thus increase the risk of turning users into automatic re-enactors of cultural and social norms, potentially diminishing their agency and responsibility for making and accounting for their own decisions (55).

### 4.3 Agency, persuasion, and safety

Within these collaborative arrangements, a fundamental issue becomes the allocation of control between human actors and AI systems, particularly regarding the extent to which users retain decision-making authority (56). Zhang et al. (57) identify multiple forms through which AI agency can manifest. In particular, proactive agency refers to situations where AI systems autonomously contribute recommendations or initiate actions. These situations call for the design of systems that preserve user autonomy, for example by allowing users to determine the desired level of system proactivity and adjust the frequency and type of recommendations received.

Exercise recommendations, however, may entail concrete risks, particularly for people with chronic diseases, functional limitations, cardiac rehabilitation needs, or other health vulnerabilities, such as the risk of overtraining. As theoretical literature suggests, these risks may be further amplified when a high level of proactivity allows LLM-based conversational agents to subtly influence users by framing recommendations as friendly advice from “a companion”, when in fact such recommendations may be intrinsically authoritative (58).

From a safety perspective, the deployment of LLM-based conversational agents should incorporate expert validation mechanisms to ensure that exercise recommendations are aligned with established clinical and public health guidelines, while ensuring appropriate human oversight in higher-risk situations. These systems have important limitations as automated coaches, particularly when individualized exercise prescriptions require consideration of medical history or potential contraindications. Consequently, LLM-based conversational agents should be positioned as tools that complement rather than replace professional assessment and clinical decision-making.

### 4.4 Anthropomorphization and ethical risks

Another concern directly refers to the high degree of anthropomorphization that LLM-based conversational agents seem to elicit in their users, much more than any previous conversational technology (59). Not only do people tend to ascribe human-like qualities to this technology, but it may also be purposefully designed to exhibit anthropomorphized traits (51).

However, although these systems can convincingly display behaviors associated with empathy, affect, and social intelligence, such qualities are ultimately simulated rather than genuinely possessed, as LLMs lack any form of emotional awareness or subjective experience (60). Even so, many participants across the reviewed studies frequently interpreted LLMs’ conversational cues as evidence of underlying emotional or social capacities. This tendency to ascribe human-like qualities may stem from the fact that anthropomorphic explanations provide an intuitive and readily accessible way for users to make sense of the system's behavior, especially when confronted with complex interactional cues, as suggested by the literature analyzing users’ perceptions of LLMs and Generative AI (43, 61). This literature further highlights that, in light of the opaque nature of LLMs’ internal processes, construing their outputs as expressions of human-like qualities can feel more reassuring than attempting to interpret them as the result of abstract and largely inaccessible computational mechanisms (61).

However, attributing emotions, empathy, or social competence to LLMs can generate problematic consequences. Theoretical literature points out that users may develop unrealistic expectations about the system's capabilities, or even feel a misplaced sense of obligation toward it, investing time, effort, or emotional energy in ways that are not warranted, as emphasized by previous research (48). Furthermore, responses that appear supportive or caring can foster the illusion of a reciprocal relationship, while concealing the extent to which outputs are shaped by hidden training data, design decisions, and institutional agendas (62). This framing risks presenting interactions as inherently benign, diverting attention from the ways in which LLMs may reproduce and reinforce historically situated forms of bias (63).

These risks call for the design of interactions that limit excessive anthropomorphic cues and make the system's limitations explicit. For example, conversational agents should clearly communicate that they do not possess emotions, intentions, or genuine social understanding, despite their ability to simulate such qualities through natural language.

### 4.5 Limitations and strengths

This review has several limitations. Firstly, it includes a small number of studies, which are heterogeneous in terms of designs and outcomes. Secondly, we relied on two databases, while the exclusion of other databases may have reduced the coverage of the review: relevant studies indexed in discipline-specific databases, such as PubMed/MEDLINE, IEEE Xplore, PsycINFO, or CINAHL, may have been missed. Thirdly, the screening and extraction of included papers were conducted by only one reviewer, potentially increasing the risk of selection bias, extraction errors, and subjective interpretation. Furthermore, quality appraisal was conducted by a single reviewer, which may increase the risk of subjective judgment in the assessment of study robustness. Relatedly, the search strategy was not peer-reviewed by an information specialist, which may have affected its sensitivity.

The choice of including only articles published in international conferences and journals may also lead to the potential exclusion of relevant grey literature or preprints. By contrast, this review also has several strengths. First, it addresses a highly relevant topic, given the increasing importance and diffusion of LLMs in behavioral interventions, focusing on an emerging field. Second, it integrates experiential, behavioral, relational, and ethical dimensions of the use of LLMs in physical activity support. Finally, we attempted to move beyond purely technical evaluations of LLM-based conversational agents, addressing their experiential effects.

### 4.6 Conclusion and future perspectives

This review pointed out that while LLM-based conversational agents appear promising for supporting engagement and reflection, the evidence remains insufficient to conclude that they have a sustained effect on objectively measured physical activity. Moreover, it highlighted that LLM-based conversational agents may be designed to assume specific social roles, and that these roles may shape how people relate to them. Finally, it emphasized that these technologies may trigger the ascription of human-like qualities.

Given the limitations of the studies analyzed in this review, future research should prioritize longitudinal studies conducted in real-world settings evaluating long-term adherence. Moreover, research should focus on collecting objective measures of physical activity, using stronger comparators, to assess whether the proposed interventions are effective in changing the target behavior. To increase the generalizability and reproducibility of the reported results, researchers should involve more diverse populations and promote more rigorous reporting of model characteristics, prompts, and implementation procedures. At the same time, greater attention should be devoted to safety evaluations, particularly regarding the appropriateness of exercise recommendations, the risks associated with over-reliance on automated coaching, and the role of human oversight in higher-risk situations. Finally, further work is needed to examine how relationships with LLM-based conversational agents evolve over time and how anthropomorphization may influence the outcomes of the interventions.

## Author contributions

AS: Writing – review & editing, Investigation, Writing – original draft, Conceptualization, Data curation, Formal analysis, Methodology. AB: Writing – review & editing. MC: Writing – review & editing. AR: Project administration, Writing – review & editing, Supervision, Methodology, Writing – original draft, Conceptualization, Investigation, Funding acquisition, Validation.

## Footnotes

^1^
See https://osf.io/tr7gy/overview?view_only=a8225f67eb0c4ffe8051bfe8a0934160, last accessed June 2026.
